# Granulocyte-macrophage stimulating factor (GM-CSF) increases circulating dendritic cells but does not abrogate suppression of adaptive cellular immunity in patients with metastatic colorectal cancer receiving chemotherapy

**DOI:** 10.1186/1475-2867-12-2

**Published:** 2012-01-23

**Authors:** Micaela Martinez, Nadia Ono, Marina Planutiene, Kestutis Planutis, Edward L Nelson, Randall F Holcombe

**Affiliations:** 1 Division of Hematology/Oncology, University of California, Irvine, CA, USA; 2 Tisch Cancer Institute of Mt. Sinai School of Medicine, New York, NY, USA

## Abstract

**Background:**

Advanced cancer and chemotherapy are both associated with immune system suppression. We initiated a clinical trial in patients receiving chemotherapy for metastatic colorectal cancer to determine if administration of GM-CSF in this setting was immunostimulatory.

**Methods:**

Between June, 2003 and January, 2007, 20 patients were enrolled in a clinical trial (NCT00257322) in which they received 500 ug GM-CSF daily for 4 days starting 24 hours after each chemotherapy cycle. There were no toxicities or adverse events reported. Blood was obtained before chemotherapy/GM-CSF administration and 24 hours following the final dose of GM-CSF and evaluated for circulating dendritic cells and adaptive immune cellular subsets by flow cytometry. Peripheral blood mononuclear cell (PBMC) expression of γ-interferon and T-bet transcription factor (*Tbx21*) by quantitative real-time PCR was performed as a measure of Th1 adaptive cellular immunity. Pre- and post-treatment (i.e., chemotherapy and GM-CSF) samples were evaluable for 16 patients, ranging from 1 to 5 cycles (median 3 cycles, 6 biologic sample time points). Dendritic cells were defined as lineage (-) and MHC class II high (+).

**Results:**

73% of patients had significant increases in circulating dendritic cells of ~3x for the overall group (5.8% to 13.6%, p = 0.02) and ~5x excluding non-responders (3.2% to 14.5%, p < 0.001). This effect was sustained over multiple cycles for approximately half of the responders, but tachyphylaxis over subsequent chemotherapy cycles was noted for the remainder. Treatment also led to a significant reduction in the proportion of circulating regulatory T-cells (Treg; p = 0.0042). PBMC *Tbx21 *levels declined by 75% following each chemotherapy cycle despite administration of GM-CSF (p = 0.02). PBMC γ-interferon expression, however was unchanged.

**Conclusions:**

This clinical trial confirms the suppressive effects of chemotherapy on Th1 cellular immunity in patients with metastatic colorectal cancer but demonstrates that mid-cycle administration of GM-CSF can significantly increase the proportion of circulating dendritic cells. As the role of dendritic cells in anti-tumor immunity becomes better defined, GM-CSF administration may provide a non-toxic intervention to augment this arm of the immune system for cancer patients receiving cytotoxic therapy.

**Trial Registration:**

ClinicalTrials.gov: NCT00257322

## Background

Tumor infiltration by cells involved in the cellular immune response appears to correlate with prognosis for patients with colon cancer, suggesting that the immune system plays a critical role in patient outcome [1-3]. Following surgical resection of a colonic tumor, improved overall survival in patients with early stage (I-III) disease correlates with the extent of immune infiltration of the primary tumor with dendritic cells and T-cells. The reduced relapse rate in these patients suggests that sensitization to tumor antigens and an associated viable cellular immune response may eradicate microscopic residual disease. While patients with stage IV (metastatic) colorectal cancer are generally considered incurable, it is theoretically possible that stimulation of anti-tumor aspects of the immune system might positively affect response to therapy and overall median survival [4], analogous to the phenomenon noted for earlier stages of this disease. In addition, patients with advanced cancer are known to have tumor-related immune suppression and chemotherapy, typically administered in this setting, is also immunosuppressive [5,6]. However, the degree of immunosuppression of different chemotherapy regimens, and the precise effects of chemotherapy on specific lymphocyte subsets have not been rigorously defined. Still, for patients with colon cancer, reversing the innate immune suppression caused by any of the potential etiologies may be advantageous.

GM-CSF is frequently utilized in cancer patients to aid in hematopoietic recovery but it also has pleiotropic effects on components of the immune system, including being a requisite growth factor for myeloid dendritic cells (DCs) that play an important role in tumor antigen presentation and stimulation of immune responses [7]. Our hypothesis was that GM-CSF would be beneficial for patients with advanced colorectal cancer by stimulating cellular immune responses, potentially including Th1 immune responses, specifically by activating dendritic cells and also might abrogate chemotherapy induced immunosuppression common in this patient population. We devised a phase II trial augmenting standard chemotherapy with mid-cycle GM-CSF and monitored effects on the circulating dendritic cell and other peripheral blood mononuclear cell (PBMC) populations along with evaluation of Th1 type immune responses. The principal endpoints of this study were biologic in order to provide a potential rationale for larger studies with survival endpoints including GM-CSF.

## Materials & methods

### Clinical trial design and conduct

Patients who were receiving chemotherapy for stage IV colorectal cancer were identified for this study. Enrollment for this study began in June, 2003 and ended January 2007, with 20 patients enrolled onto clinical trial NCT00257322. The study was approved by the University of California, Irvine Institutional Review Board and all patients signed written informed consent prior to participation. All research was conducted in compliance with the Helsinki Declaration for ethical research in humans. Patients received 500 μg GM-CSF daily for 4 days starting 24 hours after completion of each chemotherapy cycle. Initial patients received weekly 5-fluorouracil with leucovorin (n = 12). With changes in standard of care that occurred during the conduct of the study, a protocol modification allowed enrollment of patients receiving biweekly mFOLFOX6 (5FU, leucovorin, oxaliplatin) chemotherapy (n = 8). Blood was obtained before chemotherapy/GM-CSF administration and 24 hours following the final dose of GM-CSF.

### Quantitative Real-time PCR (qRT-PCR)

Blood samples drawn from the patients were immediately processed for the isolation of PBMCs using Histopaque 1083 (Sigma-Aldrich). Trizol (Invitrogen) was added to the PBMC pellets and stored at 4°C for subsequent RNA extraction. cDNA preparation for each RNA sample was performed using the high capacity cDNA reverse transcription kit (Applied Biosystems). PBMC expression of γ-interferon, T-bet transcription factor (*Tbx21*), DC-LAMP, and Wnt 5a by quantitative real-time PCR was performed, the former two as measures of Th1 immunity. All experimental mRNA were normalized to the housekeeping gene β-actin mRNA.

### Flow cytometry

Standard whole blood staining protocols were used to evaluate circulating leukocyte populations. Phlebotomy samples in EDTA anti-coagulated blood were collected and individual aliquots of 200 μl of blood were placed into #2059 tubes (BD Biosciences) and stained with the appropriate experimental or isotype control antibodies. Tubes were then incubated at room temperature (RT) for 60 minutes in the dark, with mixing every 15 minutes. RBCs were lysed with ACK lysis media for 15 minutes at RT and residual cells pelleted by centrifugation. Supernatant was discarded by aspiration, cells washed and the cell pellet was resuspended in either 500 μl of staining media (if flow cytometry analysis is to be immediate) or 50% volume of staining media and 50% volume freshly prepared 4% paraformaldehyde. In the latter case tubes were stored at 4°C in the dark for no more than 48 hours before flow cytometry analysis. Calabrite 3, a calibration bead set (BD Biosciences) were used for standardization of the conditions and compensation controls for the three-color flow cytometry analysis according to manufacturer's instructions. Additional fluorophore controls, CD3-FITC, CD8-PE, CD4-TC, were used to verify the uniformity of conditions for the analyses.

### Statistical Methods

Expression of mRNA by real-time PCR was compared, pre- and post-treatment, with a Wilcoxin matched pairs signed rank test. Changes in lymphocyte subsets pre- and post-treatment were compared similarly. Differences across cycles of chemotherapy/GM-CSF administration were analyzed for statistical significance by a two-tailed unpaired t-test with Welch's correction.

## Results

### Clinical trial

Twenty patients were enrolled in the clinical trial and pre- and post-treatment (i.e., chemotherapy and GM-CSF) samples were evaluable for 16 patients. Samples were obtained ranging from 1 to 5 cycles with a median of 3 cycles (6 biologic sample time points) overall. There were no toxicities or adverse events to GM-CSF reported. Initially, most patients received weekly 5FU/leucovorin though later, because of changes that occurred in the standard of care, the protocol was amended to include patients receiving biweekly mFOLFOX6. No significant differences in immunologic endpoints were seen between the two groups. In addition, no patients were removed for study for hematologic toxicities and there was no increased toxicity beyond that seen in other trials with these specific chemotherapy agents without GMCSF. In all cases, patients received 4 daily dosages of GM-CSF starting 24 hours after completion of the chemotherapy cycle.

### Cellular immune response

**Figure 1:**
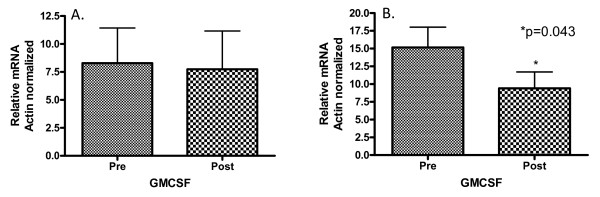
**Panel A. Levels of γ-interferon mRNA following the administration of GM-CSF after chemotherapy**. γ-interferon mRNA levels were measured in 16 patients pre-chemotherapy/GM-CSF administration and post-GM-CSF/chemotherapy administration by quantitative real time PCR of peripheral blood mononuclear cells. γ-interferon mRNA levels were normalized against the housekeeping gene β-actin mRNA. There was no statistically significant change between the two groups. Panel B. Levels of T-bet transcription factor mRNA following the administration of GM-CSF after chemotherapy. T-bet transcription factor mRNA levels were measured in 16 patients pre-chemotherapy/GM-CSF administration and post-GM-CSF/chemotherapy administration by quantitative real time PCR of peripheral blood mononuclear cells. T-bet transcription factor mRNA levels were normalized against the housekeeping gene β-actin mRNA. There was an approximate 1.7 fold decrease in T-bet transcription factor mRNA levels following the administration of GM-CSF after chemotherapy (p = 0.043). The statistical significance was determined by a Wilcoxin matched pairs test.

Cellular immune responses were quantified through the evaluation of expression of relevant genes from PBMCs by qRT-PCR. Expression of mRNA for both DC-lamp, a transmembrane glycoprotein expressed on mature dendritic cells [8] and Wnt5a, which activates macrophages and T-cells through its interaction with the cell surface frizzled-5 receptor [9] were minimally detected in the patient samples (Figures not shown). γ-interferon mRNA levels remained unchanged with the administration of GM-CSF after chemotherapy (Figure 1A). γ-interferon is produced by Th1 cells, NK cells and, to a lesser extent, dendritic cells [10]. However, there was a statistically significant 1.7 fold decrease in T-bet transcription factor (*Tbx21*) mRNA following the administration of GM-CSF after chemotherapy (p = 0.043; Figure 1B), implicating an overall decrease in Th1-type cellular immune response [11].

### Lymphocyte subsets and dendritic cells

**Figure 2:**
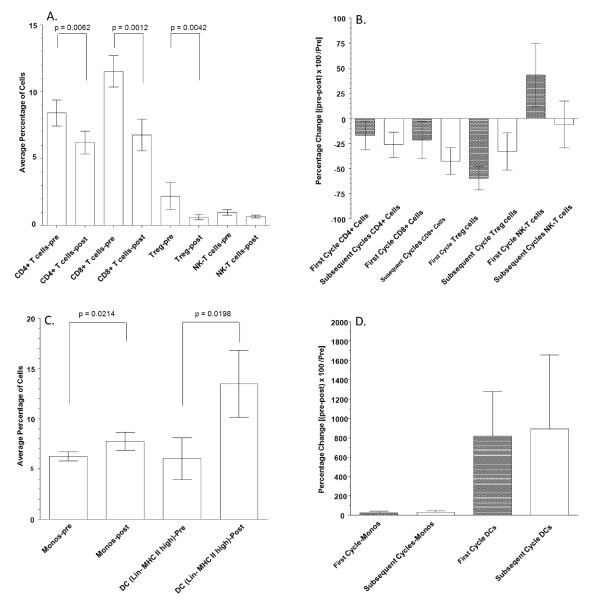
**Panel A. Changes in lymphocyte subsets associated with chemotherapy and GM-CSF administration**. Individual lymphocyte subsets were evaluated by flow cytometry before and after the administration of chemotherapy and GM-CSF. The subsets that were evaluated include: CD4+, CD8 +, CD4+CD25 high (Treg), CD56+ CD3+ (NK-T) populations. Values depicted represent average percentages of nucleated cells in whole blood samples (n = 23) with error bars depicting standard error values. Significant differences were evaluated with a Wilcoxon matched pairs test. The decrease in CD4 and CD8 T lymphocyte subsets was statistically significant at p = 0.0064 and p = 0.0012 respectively. The decrease in the Treg population was also significant at p = 0. 0042 while the decrease in NK-T cells approached statistical significance p = 0.0997. Panel B. Percent change in lymphocyte subsets across multiple cycles of chemotherapy & GM-CSF. In the first and subsequent cycles of Chemotherapy & GM-CSF the percent change in lymphocyte subsets that include: CD4+, CD8 +, CD4+CD25 high (Treg), CD56+ CD3+ (NK-T) populations, were determined. Percent change was calculated in the standard fashion [(pre- treatment value - post-treatment value)/pre-treatment value] × 100. None of the differences in lymphocyte subset changes between the first and subsequent cycles reached statistical significance by two-tailed unpaired t-test with Welch's correction. Panel C. Changes in monocytes and myeloid DCs associated with chemotherapy and GM-CSF administration. Individual cell populations were evaluated by flow cytometry before and after the administration of chemotherapy and GM-CSF. Monocyte populations were identified as being CD14+. Myeloid dendritic cells (DCs) were identified using a lineage cocktail (CD3, CD20, CD14, CD56) negative cells that had MHC II high expression. Values depicted represent average percentages of nucleated cells in whole blood samples (n = 23) with error bars depicting standard error values. Differences were evaluated with a Wilcoxon matched pairs test. Differences in monocytes and DCs were statistically significant p = 0.0214 and p = 0.0198 respectively. Panel D. Percent change in monocytes and dendritic cells across multiple cycles of chemotherapy and GM-CSF. In the first and subsequent cycles of Chemotherapy & GM-CSF the percent change in monocytes (CD14+ cells) and dendritic cells (DCs) [lineage cocktail (CD3, CD20, CD14, CD56) negative, MHC II high expression] were determined. Percent change was calculated in the standard fashion [(pre-treatment value - post-treatment value)/pre-treatment value] × 100. None of the differences in lymphocyte subset changes between the first and subsequent cycles reached statistical significance by two-tailed unpaired t-test with Welch's correction.

Individual lymphocyte subsets were evaluated by flow cytometry before and after the administration of chemotherapy and GM-CSF. The subsets that were evaluated include: CD4+, CD8 +, CD4+CD25 high (Treg) and CD56+ CD3+ (NK-T) populations. Values represent average percentages of nucleated cells in whole blood samples (n = 23). Significant differences were evaluated with a two-tailed Wilcoxon Signed rank test. The decrease in CD4 and CD8 T lymphocyte subsets was statistically significant at p = 0.0064 and p = 0.0012 respectively. The decrease in the Treg population was also significant at p = 0.0042 and the decrease in NK-T cells approached statistical significance of p = 0.0997 (Figure 2A).

In the first and subsequent cycles of chemotherapy and GM-CSF the percent change in lymphocyte subsets that include CD4+, CD8 +, CD4+CD25 high (Treg) and CD56+ CD3+ (NK-T) populations were determined. Percent change was calculated in the standard fashion [(Pre treatment value-Post treatment value)/pre-treatment value] × 100. None of the differences in lymphocyte subset changes between the first and subsequent cycles reached statistical significance by two-tailed unpaired t-test with Welch's correction (Figure 2B).

Individual cell populations were evaluated by flow cytometry before and after the administration of chemotherapy and GM-CSF. Monocyte populations were identified as being CD14+. Myeloid DCs were identified using a lineage cocktail (CD3, CD20, CD14, CD56) negative cells that had MHC II high expression. Values represent average percentages of nucleated cells in whole blood samples (n = 23). There were statistically significant increases in both monocytes (p = 0.0214) and DCs (p = 0.01980) following chemotherapy and GM-CSF (Figure 2C). Overall, there was no difference between the first and subsequent cycles of chemotherapy and GM-CSF in the percent change in monocytes (CD14+ cells) and DCs (lineage cocktail (CD3, CD20, CD14, CD56) negative, MHC II high expression; Figure 2D). However, approximately 50% of the patients exhibited increased dendritic cell numbers in subsequent chemotherapy/GM-CSF cycles.

## Discussion

GM-CSF is utilized primarily for its activity in increasing granulocyte production following administration of bone marrow suppressive cytotoxic chemotherapy. However, largely because of its role in stimulating monocytic precursors, it has long been recognized that it possesses immunostimulatory activity [7]. Our utilization of GM-CSF in this trial was to attempt to abrogate the immunosuppression induced by chemotherapy [5] and by the cancer itself [6]. In this population of patients receiving chemotherapy for metastatic colorectal cancer, GM-CSF was administered without significant toxicity.

GM-CSF did not augment cellular, Th1, immune functioning, as measured by the methodologies utilized in this study. The reduction in T-bet is postulated to be secondary to chemotherapy. The lack of concomitant reduction of γ-interferon may be due to GM-CSF-dependent stimulation of dendritic cells, which can also produce this cytokine. Together with the decreases in the CD4+, CD8+, CD14+ and CD56 =/CD3+ populations, the decrease in T-bet suggests that there was not selective sparing of Th1 biased lymphocytes by the cytotoxic chemotherapy to account for the stable γ-interferon mRNA expression in PBMCs. As there was no control arm of patients receiving chemotherapy without GM-CSF on this study, we cannot quantitate the absolute reduction in PBMC γ-interferon production which might be seen following chemotherapy alone. With respect to T-cell subset changes, approximately 2/3 (66%) of subjects had a significant decrease in CD4+ lymphocytes and approximately 80% of subjects had a significant decrease in CD8+ lymphocytes with each cycle.

One of the most significant findings from this clinical trial was the increase in the number of circulating myeloid DCs (Lineage-, MHC high). These increased in approximately 75% of subjects across all cycles. In subsequent cycles of chemotherapy and GM-CSF, approximately 50% of the subjects experienced increased DC numbers over the initial cycle. Dendritic cells are potent antigen presenting cells and play a pivotal role in the induction of immune responses. Dendritic cells have been directly implicated in anti-tumor immune responses [12-15]. The increase in circulating DCs, likely a consequence of GM-CSF administration, may contribute to the anti-tumor effects of chemotherapy administered in the adjuvant or metastatic setting.

Another important observation was the reduction in regulatory T-cells (Treg) following chemotherapy/GM-CSF. Approximately 90% of subjects had a significant (> 20%) decrease in Treg lymphocytes with the first cycle. Tregs can suppress the activity of cytotoxic T cells through direct cell-to-cell contact or via the release of cytokines such as transforming growth factor beta. Depletion of intratumoral Tregs has been shown to enhance antitumor immunity and promote tumor rejection in mouse models [16]. In patients with colon cancer, increased numbers of Treg cells has been associated with a poor clinical prognosis [17]. This decrease in circulating Treg cells argues against a significant increase in myeloid derived suppressor cells, implicated in the induction of Tregs [18], as a result of the administration of GM-CSF; an effect that has been inconsistently reported in other settings[19-22]. Thus, the reduction in Tregs seen in our study, and the concomitant loss of immune system suppression that these cells induce, may be prognostically favorable.

As noted previously, this study did not contain a chemotherapy only control arm. Therefore, the effects of chemotherapy cannot be distinguished specifically from the effects of GM-CSF. Still, in patients with metastatic colorectal cancer receiving chemotherapy and GM-CSF, cellular immune responses are inhibited and the impairment in Th1 type immune parameters are not abrogated by administration of GM-CSF. However, administration of GM-CSF along with chemotherapy does lead to an increase in circulating DCs and a reduction in inhibitory Tregs. Together, this may provide a mechanism for augmentation of innate immune responses and may provide benefit to colorectal cancer patients receiving chemotherapy.

## Conclusions

This phase II clinical trial confirms that chemotherapy in patients with metastatic colorectal cancer suppresses Th1 cellular immunity but demonstrates that mid-cycle administration of GM-CSF can significantly increase the proportion of circulating dendritic cells and reduce the proportion of circulating Treg cells. Th1 activity, as measured by γ-interferon production, was not affected. These finding suggest that GM-CSF can abrogate important aspects of the immune suppression seen in patients with advanced colorectal cancer and may provide a non-toxic intervention to augment anti-tumor immunity for cancer patients receiving cytotoxic chemotherapy.

## Competing interests

The authors declare that they have no competing interests.

## Authors' contributions

MM, NO, MP and KP conducted immunologic assays including PCR and flow cytometry. MM assisting in maintaining clinical trial related data according to IRB guidelines. ELN and RFH conceived this study, provided oversight for all aspects of the laboratory investigation, were involved in obtaining informed consent and providing treatment for patients and directed the overall project. All authors were involved in manuscript preparation.
