# Correction: Granulocyte-macrophage stimulating factor (GM-CSF) increases circulating dendritic cells but does not abrogate suppression of adaptive cellular immunity in patients with metastatic colorectal cancer receiving chemotherapy

**DOI:** 10.1186/1475-2867-13-80

**Published:** 2013-08-15

**Authors:** Miceala Martinez, Nadia Ono, Marina Planutiene, Kestutis Planutis, Edward L Nelson, Randall F Holcombe

**Affiliations:** 1 Division of Hematology/Oncology, University of California, Irvine, CA, USA; 2 Tisch Cancer Institute of Mt. Sinai School of Medicine, New York, NY, USA

## Correction

After publication of the original article [1] it came to the publishers attention that the article was incorrectly published as an Editorial instead of the correct article type; Primary Research. We apologise for any inconvenience this has caused.
